# Supplementary material for: Mini-bacterioferritins: structural insight into a ferritin-like protein from the anaerobic methane-oxidising archaeon Candidatus Methanoperedens carboxydivorans
Source: Commun Biol. 2026 Mar 21;9:646. doi: 10.1038/s42003-026-09796-4 (PMC13168243; doi:10.1038/s42003-026-09796-4)
Supplement: Supplementary file 2 — Supplementary information [file 42003_2026_9796_MOESM2_ESM.pdf]

Supplementary information to

Mini-bacterioferritins: Structural insight into a ferritin-like protein from the anaerobic methane-oxidising archaeon *Candidatus* Methanoperedens carboxydivorans

Martijn Wissink[1], Sylvain Engilberge[2], Pedro Leão[1], Robert S. Jansen[1], Mike S. M. Jetten[1], Mélissa Belhamri[2], Olivier N. Lemaire[2], Antoine Royant[2,3], Cornelia U. Welte[1]*, Tristan Wagner[2,4]*

[1] Department of Microbiology, Radboud Institute for Biological and Environmental Sciences, Radboud University, Heyendaalseweg 135, 6525AJ Nijmegen, the Netherlands

[2] Univ. Grenoble Alpes, CEA, CNRS, Institut de Biologie Structurale, 38044, Grenoble, France.

[3] European Synchrotron Radiation Facility, 38043, Grenoble, France

[4] Microbial Metabolism Research Group, Max Planck Institute for Marine Microbiology, Celsiusstraße 1, 28359 Bremen, Germany

*Address correspondence to Cornelia U. Welte, E-mail address: c.welte@science.ru.nl and Tristan Wagner, E-mail address: twagner@mpi-bremen.de

Summary: 8 pages, 7 figures. Supporting data of main text figures and tabulated supplementary data can be found in the Excel file Supplementary Data.

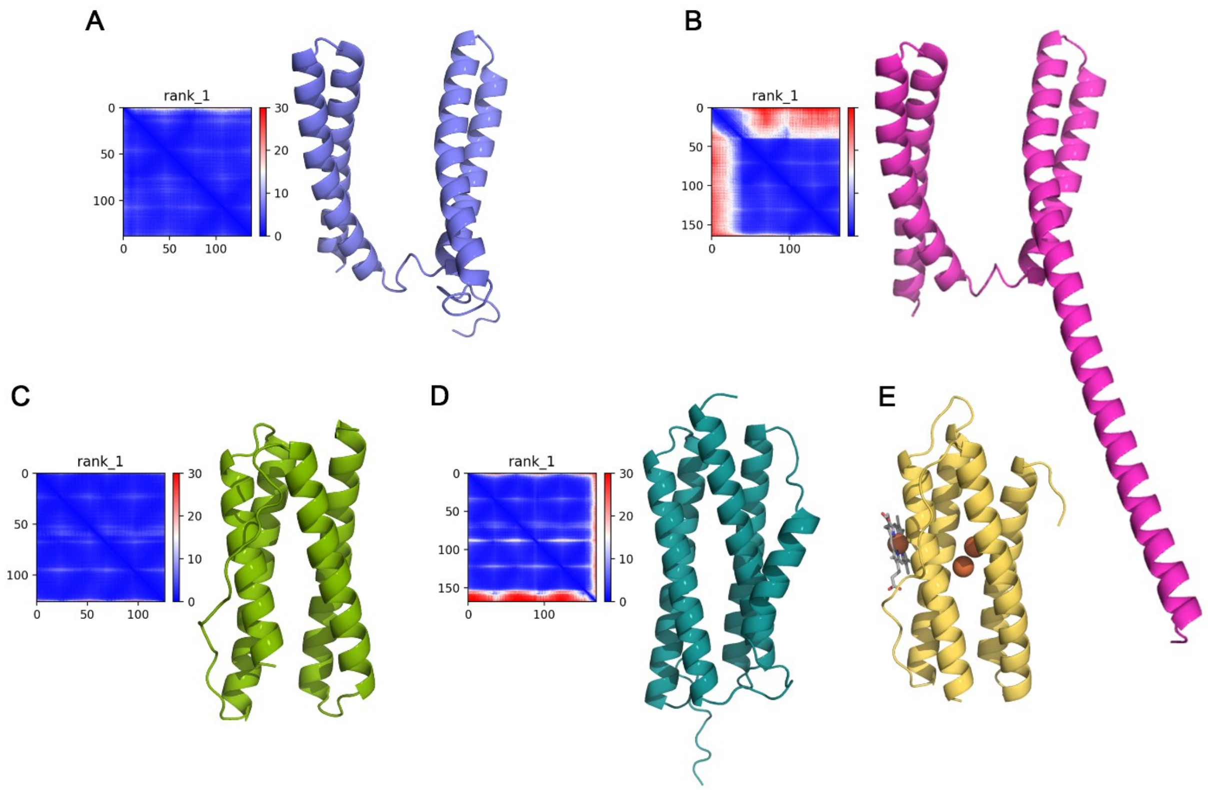

Supplementary Figure 1. AlphaFold models of ferritin-like proteins identified in '*Ca*. M. carboxydivorans'. Each panel displays the Predicted Aligned Error (PAE) plot alongside the corresponding protein model in cartoon representation. (A) WP_097297487.1 (UniProt accession A0A6A2G1C8). (B) WP_176505107.1 (UniProt accession UPI0015968CAC). (C) WP_217993135.1 (UniProt accession A0A0P7ZGQ0). (D) WP_097298589.1 (UniProt accession A0A822J9T9). (E) Cartoon representation of *Mper*-mBfr.

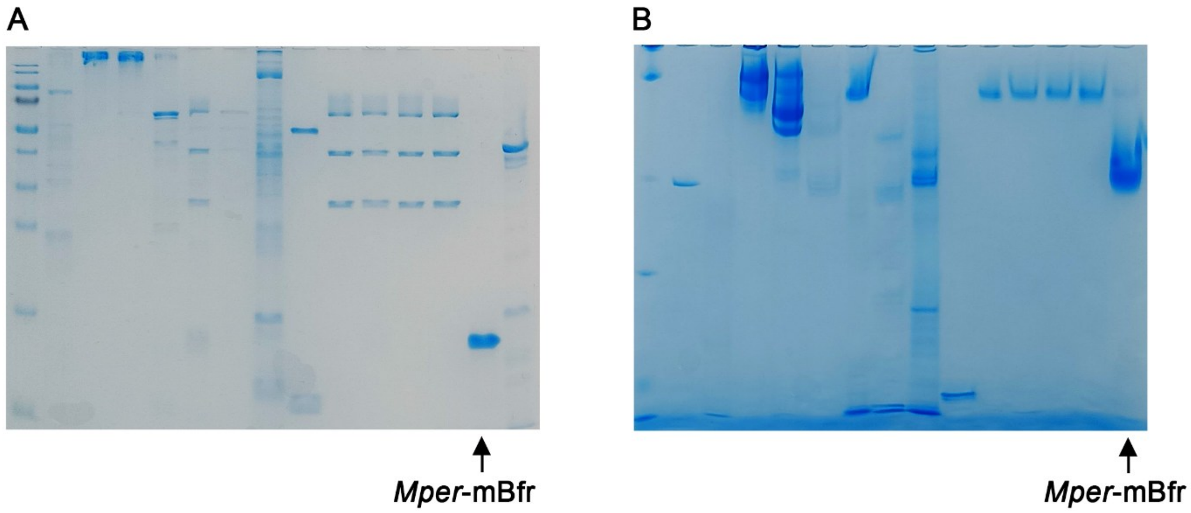

Supplementary Figure 2. Uncropped denaturing SDS-PAGE (A) and hrCN-PAGE (B) gel images of *Mper*-mBfr.

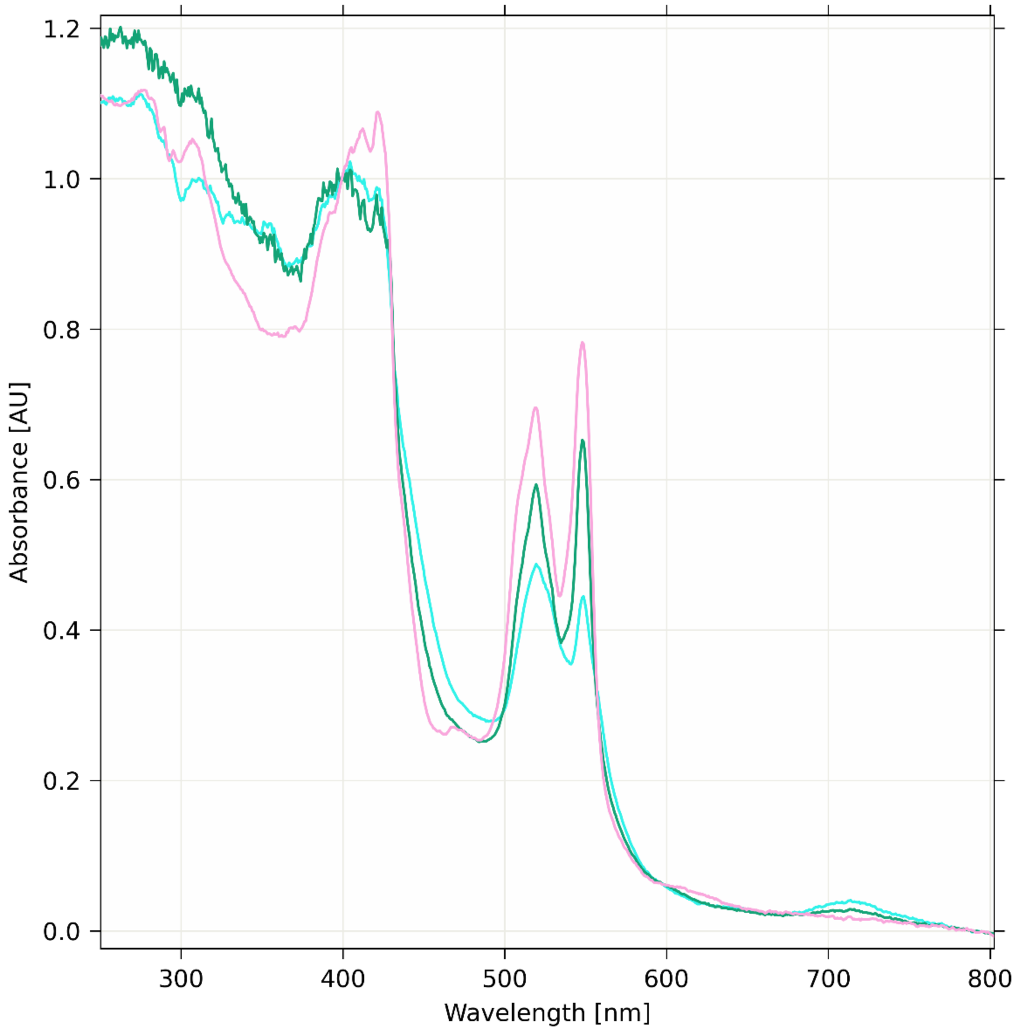

Supplementary Figure 3. Complete spectra of *Mper*-mBfr *in crystallo.* *In crystallo* spectra of the as-isolated crystal (pink), partially oxidised upon 10 minutes O_2 exposure (cyan), and oxidised upon 10 minutes O_2 exposure (green), then reduced by soaking the crystals for 4 min 40 sec in 100 mM sodium dithionite. Spectra are shown with applied baseline correction.

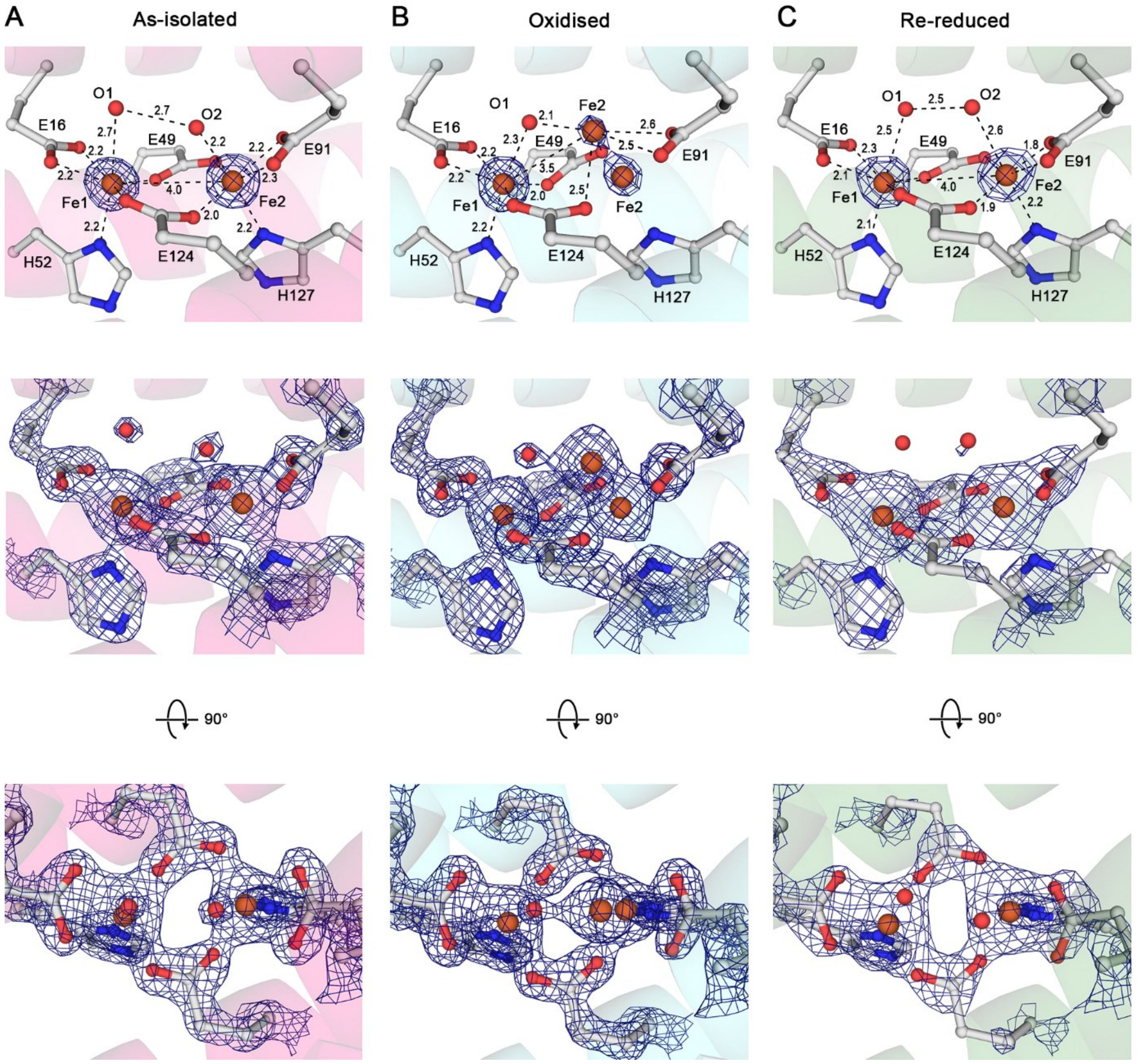

Supplementary Figure 4. Redox cycling of *Mper*-mBfr crystals. Zoom-in of the active site of the as-isolated (A), partially oxidised upon 10 minutes O_2 exposure (B), and partially oxidised upon 10 minutes O_2 exposure then reduced by soaking the crystals for 4 min 40 sec in 100 mM sodium dithionite (C) conditions. The top panel displays the distances between ligands in Å using dotted lines. The $2F_o{-}F_c$ map is contoured around the iron atoms to 7 σ as a dark blue mesh. The middle panel displays the same view, but with the $2F_o{-}F_c$ map as a dark blue mesh contoured to 2.5 σ around the iron atoms, the coordinating ligands, and the water molecules. The bottom panel displays a 90° rotation along the x-axis of the middle panel. Ligands are represented as balls and sticks, with carbon, oxygen, nitrogen, and iron coloured in white, red, dark blue, and orange, respectively.

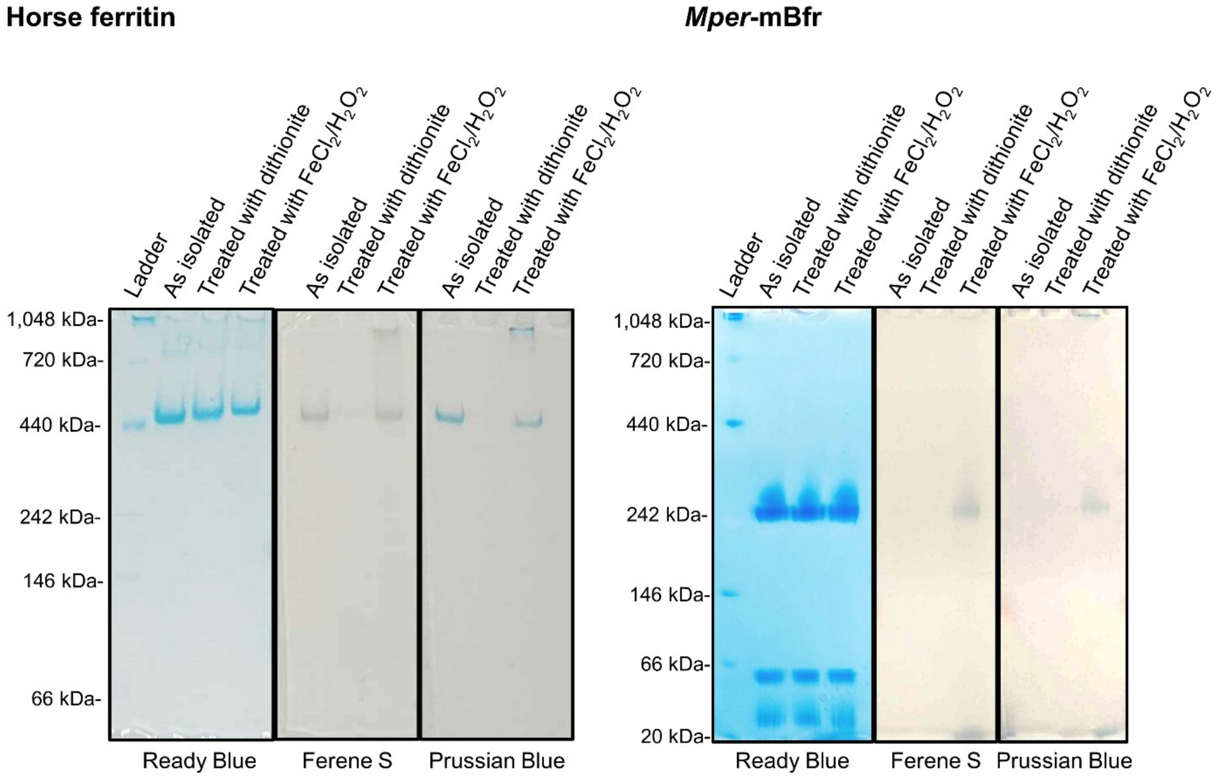

Supplementary Figure 5. Ferritin activity and Fe incorporation. Commercial horse ferritin and enriched *Mper*-mBfr were incubated for 45 minutes at 20 °C anaerobically in the presence of dithionite or Fe(II)Cl_2/dithionite/H_2O_2 to assay their capacity for internalising Fe. 4.5 µg of protein were loaded on hrCN PAGE. After migration, the gel was cut into three parts. Each part was stained with a specific solution: Ready Blue to detect all proteins, Ferene S to detect Fe, and Prussian Blue to detect Fe(III). *Mper*-mBfr enrichment protocol via ammonium sulfate precipitation resulted in slight contamination, as evidenced by two additional bands between 20 and 66 kDa.

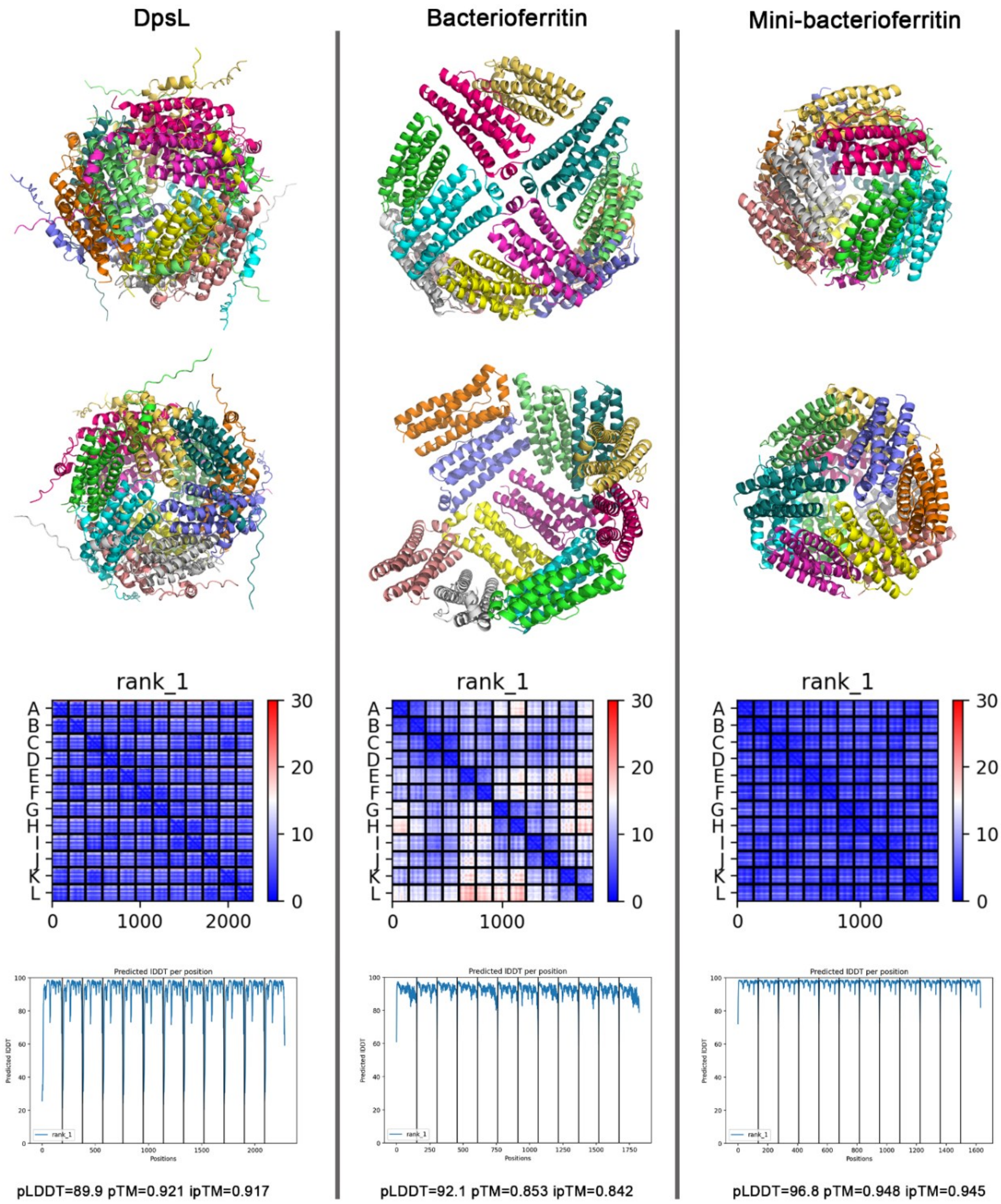

Supplementary Figure 6. Protein model representatives. Protein models were generated using ColabFold with 12 copies as input to classify proteins in the phylogenetic tree (Fig. 7). This figure presents the three predicted types of ferritins: DpsL (left) from *Stenotrophomonas sp*. (UniProt accession code A0A3Q8FCL6), bacterioferritin (middle) from *Deltaproteobacteria* bacterium (UniProt accession code A0A0S8HYC1), and mini-bacterioferritin (right) from '*Candidatus* Scalindua rubra' (UniProt accession code A0A1E3X7Q7). From top to bottom, the models are shown in two different orientations using a cartoon representation, followed by the Predicted Aligned Error (PAE) plot and the per-residue pLDDT confidence score plot.

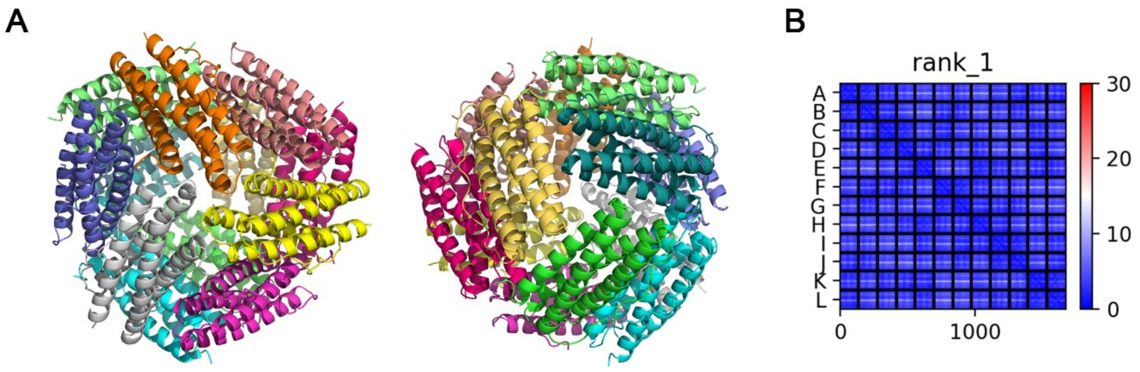

Supplementary Figure 7. Protein model of '*Ca.* Methanoperedens sp.' Vercelli mini-bacterioferritin. (A) View of the protein from the C-terminal threefold axis (left) and N-terminal threefold axis (right). (B) Predicted Aligned Error (PAE) plot of the model.
