# Supplementary material for: Mini-bacterioferritins: structural insight into a ferritin-like protein from the anaerobic methane-oxidising archaeon Candidatus Methanoperedens carboxydivorans
Source: Commun Biol. 2026 Mar 21;9:646. doi: 10.1038/s42003-026-09796-4 (PMC13168243; doi:10.1038/s42003-026-09796-4)
Supplement: Supplementary file 3 — Description of Additional Supplementary Files [file 42003_2026_9796_MOESM3_ESM.pdf]

Description of Additional Supplementary Files

File name: Supplementary Data
Description: Large tabulated supplementary data and source data behind graphs in the main text.
